# Lipophilicity and ADMET Analysis of Quinoline-1,4-quinone Hybrids

**DOI:** 10.3390/pharmaceutics15010034

**Published:** 2022-12-22

**Authors:** Monika Kadela-Tomanek, Maria Jastrzębska, Elwira Chrobak, Ewa Bębenek

**Affiliations:** 1 Department of Organic Chemistry, Faculty of Pharmaceutical Sciences in Sosnowiec, Medical University of Silesia, 4 Jagiellońska Str., 41-200 Sosnowiec, Poland; 2 Silesian Center for Education and Interdisciplinary Research, Institute of Physics, University of Silesia, 75 Pułku Piechoty 1a, 41-500 Chorzów, Poland

**Keywords:** lipophilicity, quinoline, ADMET, molecular docking

## Abstract

Lipophilicity is one of the basic properties of a potential drug determining its solubility in non-polar solvents and, consequently, its ability to passively penetrate the cell membrane, as well as the occurrence of various pharmacokinetic processes, including adsorption, distribution, metabolism, excretion, and toxicity (ADMET). Heterocyclic compounds containing a nitrogen atom play a significant role in the search for new drugs. In this study, lipophilicity as well as other physicochemical, pharmacokinetic and toxicity properties affecting the bioavailability of the quinolone-1,4-quinone hybrids are presented. Lipophilicity was determined experimentally as well as theoretically using various computer programs. The tested compounds showed low values of experimental lipophilicity and its relationship with the type of 1,4-quinone moiety. Introduction of the nitrogen atom reduced the lipophilicity depending on the position at the 5,8-quinolinedione moiety. The bioavailability of the tested compounds was determined in silico using the ADMET parameters. The obtained parameters showed that most of the hybrids can be used orally and do not exhibit neurotoxic effects. Similarity analysis was used to examine the relationship between the ADMET parameters and experimental lipophilicity. The ability of hybrids to interact with biological targets was characterized by global reactivity descriptors. The molecular docking study showed that the hybrids can inhibit the BCL-2 protein.

## 1. Introduction

Drug design is a complex process involving identification of a molecular target, elaboration and synthesis of a new substance, and in vitro and in vivo biological testing. The first attempts to correlate pharmaceutical properties with biological activity were described in the 1950s. Further research led to the development of rules describing the relationship between the physicochemical properties of the compound and its distribution in biological systems. The early use of computational methods in combination with in vivo and in vitro predictions in the drug discovery process helps to reduce time, costs and number of animal experiments. For this reason, in the last decade, in silico absorption, distribution, metabolism, excretion and toxicity (ADMET) studies have played a key role in drug discovery as these properties account for the failure of about 60% of all drugs in the clinical phases [1,2,3,4,5,6,7].

One of the fundamental properties of a potential drug is its lipophilicity, which determines the solubility of the compound in nonpolar solvents. This parameter determines the ability of a substance to passively penetrate the cell membranes, which is associated with pharmacokinetic processes such as adsorption, distribution, metabolism and excretion, as well as with the toxicity of the potential drug [8,9,10]. Several methods have been described in the literature for determining experimental lipophilicity, including reversed phase-thin layer chromatography (RP-TLC), normal phase-thin layer chromatography (NP-TLC) or reversed phase-high performance liquid chromatography (RP-HPLC). The use of computational methods can be a valuable supplement to the experimental ones. According to the literature data, the calculated lipophilicity is more or less similar to the experimental one depending on the algorithm used in the calculation method [11,12,13,14,15].

Heterocyclic compounds containing a nitrogen atom play a significant role in the therapy of many diseases [16,17]. Quinoline and its derivatives are one of the most important heterocyclic compounds which have diverse biological activities, such as anticancer, antimalarial, antihypertensive, anti-inflammatory, antibiotic, antiviral, and antituberculosis [18,19,20,21].

**Figure 1:**
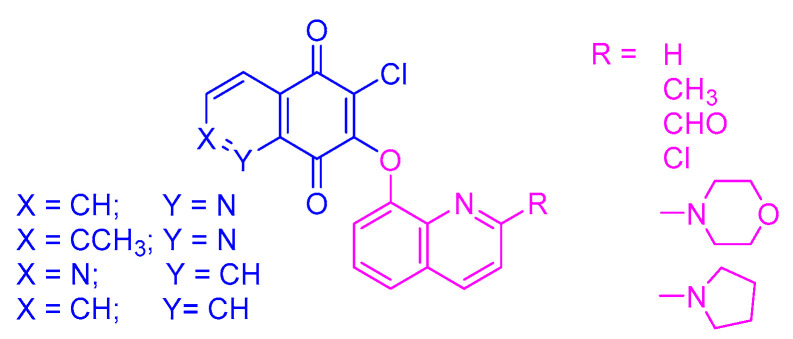
The chemical structure of quinoline-1,4-quinone hybrids.

In our earlier study, we described a new type of quinone hybrid obtained by the combination of a quinone scaffold with the 5,8-quinolinedione or 1,4-naphtoquinone moiety (Figure 1). The applied enzymatic assay showed that these compounds were good substrates of the NAD(P)H quinone dehydrogenase 1 (NQO1). The hybrids were highly active against a cancer cell line overexpressing the gene encoding the NQO1 protein. Investigation of the molecular mechanism of activity showed that the hybrids induced the mitochondrial apoptotic pathway by inhibiting the gene encoding the BCL-2 protein [22].

The aim of the present study was to determine the lipophilicity and other physicochemical, pharmacokinetic and toxicity (ADMET) properties affecting the bioavailability and biological activity of the quinoline-1,4-quinone hybrids. The analysis of the correlations between the ADMET parameters and biological activity of the hybrids was the next stage of the research. The molecular docking study was also used to examine the interaction between hybrids and BCL-2 protein.

## 2. Materials and Methods

### 2.1. Data Set

**Figure 2:**
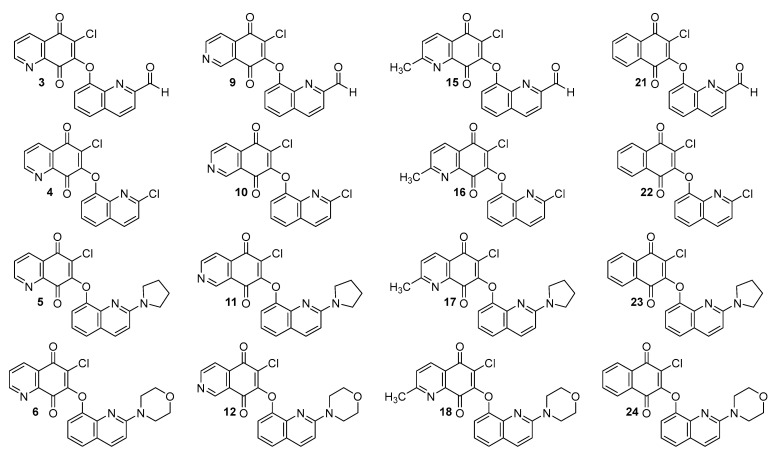
Chemical structure of hybrids **1**–**24**.

Synthesis and biological activity of the quinoline-1,4-quinone hybrids are described in the literature [22]. Their chemical structures are presented in Figure 2.

The molecular structure of the compounds **1**–**24** was optimized by the DFT (B3LYP/6-311G+++(d.p)) method implemented in the Gaussian 9.0 program package and the results were visualized using the GaussView (version 6) software package [23,24]. The obtained results are presented in Figure S1. The geometries of hybrids **1**–**24** were used to determine the molecular orbital energy, a quantum chemical descriptor.

### 2.2. Experimental Lipophilicity

The RP-TLC method was used to determine the experimental lipophilicity according to the literature [25,26,27,28]. Modified silica gel was used as the stationary phase and a mixture of tris(hydroxymethyl)aminomethane (TRIS) (0.2 M, pH = 7.4) with acetone as the mobile phase. According to our previous experiments, methanol and other polar solvents can create the hydrogen bond with the 5,8-quinolinedione moiety. For this reason, an aprotic solvent such as acetone was chosen as the mobile phase.

The amount of 5 µL of the ethanolic solution of compounds **1**–**24** and reference substance **A**–**E** was applied to the chromatographic plates using a micropipette. Each compound was tested at seven different concentrations of acetone, i.e., 50%, 55%, 60%, 65%, 70%, 75%, and 80%. Spots were visualized in iodine vapor.

### 2.3. Theoretical Lipophilicity and ADMET Parameters

The calculated lipophilicity of hybrids was determined using various online tools and free available software, including: ILOGP, XLOGP3, WLOGP, MLOGP, SILICOS-IT and milogP [29,30,31,32,33]. The ADMET parameters were determined using the pkCMS and SwissADME software [29,30,31,32].

### 2.4. Molecular Docking Study

The molecular docking study was carried out using the crystal structure of human BCL-2 protein, which was collected from the Protein Data Bank (PDB) database with the PDB identifier 4IEH [34].

The molecular docking study was performed with the AutoDock Vina software package [35]. The grid center of Vina docking was selected as the center of reference ligands, that accompanied the downloaded protein complex. The grid size was set to 14 Å × 14 Å × 14 Å, which is large enough to cover the entire target active site. Default values of all other parameters were used, and the complexes were submitted to 8 genetic algorithm runs. All obtained results were visualized using the BIOVIA Discovery Studio software package [36].

## 3. Results and Discussion

### 3.1. Experimental and Theoretical Lipophilicity

The RP-TLC method was used to evaluate the experimental lipophilicity of compounds **1**–**24** (Figure 2). The retardation parameter (R_f_) was converted to the R_M_ parameter according to Equation (1):

(1) $$R_{M}=\log\left(\frac{1}{R_{f}}-1\right)$$

The R_M_ parameter was calculated for every concentration of acetone and extrapolated to zero concentration of organic solvent in the mobile phase. The chromatographic parameter of lipophilicity (R_M0_) was calculated using Equation (2):

(2) $$R_{M}=R_{M0}+bC$$

where *C* is the concentration of acetone in the mobile phase, while *b* is the slope of the regression plot.

As seen in Table 1, the correlation coefficient r covering the range of 0.968–0.999 shows a very good correlation between the concentration of acetone and the retardation factor (R_f_).

In the next step, the relative lipophilicity parameter R_M0_ was converted to the absolute lipophilicity parameter logP_TLC_ using the calibration curve. The obtained values of the R_M0_ coefficient of the tested compounds were in the range of 1.51–4.51. The standard substances had to be selected in such a way that their literature values of log P_lit_. were within a wider range than the range of tested compounds. As reference substances, benzamide (**A**), acetanilide (**B**), 4-bromoacetophenone (**C**), benzophenone (**D**), anthracene (**E**), and dichlorodiphenyltrichloroethane (DDT) (**F**) were used, for which the literature logP_lit_ values are in the range of 0.64–6.38 [37,38]. The R_M0_ values for substances **A**–**F** were determined under the same conditions as for compounds **1**–**24**. The results are collated in Table 2.

The calibration curve Equation (3) obtained by linear correlation between the literature value of logP_lit._ and the experimental R_M0_ parameter is as follows:

logP_TLC_ = 1.1405 R_M0_ − 0.0787 (r = 0.999; SD = 0.102) (3)

Equation (3) was used to obtain the logP_TLC_ parameter for all compounds **1–24** and the results are presented in Table 3.

In general, the tested hybrids are characterized by rather low values of lipophilicity, varying in the range of 1.65–5.06. The highest values are seen for compounds **21**–**24** (logP_TLC_ in the range 4.61–5.06) containing the 1,4-naphthoquinone moiety. Introduction of the nitrogen atom reduces the lipophilicity, while the changes in its position at the 5,8-quinolinedione moiety slightly affects the logP_TLC_ parameter. According to Table 3, the trend of the values of logP_TLC_ is as follows: 5,8-quinolinedione (**1**–**6**) < 2-methyl-5,8-quinolinedione (**13**–**18**) < 5,8-isoquinolinedione (**7**–**12**). In the series of the 5,8-quinolinedione compounds (**1**–**6**), the lipophilicity depends on the type of substituent at the C-2 position of the quinone moiety with the order as follows: hydrogen atom (**1**) < carbonyl group (**3**) < methyl group (**2**) < chloride atom (**4**) < pyrrolidinyl ring (**5**) < morpholinyl ring (**6**). A similar correlation is observed for compounds with the 5,8-isoquinolinedione moiety (**7**–**12**). In the group of the 2-methyl-5,8-quinolinedione compounds (**13**–**18**), the lowest lipophilicity is observed for hybrid **14**.

Lipophilicity correlates with hydrophobicity, which determines the solubility of the compound in water [39,40]. The hydrophobicity is described by the hydrophobicity index (φ_0_), which can be calculated according to Equation (4).

(4) $$\varphi_{0}=-\frac{R_{M0}}{b}$$

If the value of the φ_0_ index is in the range of 65.88–81.13, it means that the compounds show a moderate solubility in water (Table 1). In the series of tested compounds, the hybrids with the 5,8-quinolinedione moiety (**1**–**6**) or 2-methyl-5,8-quinolinedione (**13**–**18**) possess a comparable solubility in water, varying in the range of 65.88–75.30. Compounds **1**–**6** and **7**–**12** differ in the position of the nitrogen atom on the 5,8-quinolinedione moiety. However, hybrids with the 5,8-quinolinedione moiety (**1**–**6**) show a lower value of φ_0_ index than those with the 5,8-isoquinolinedione moiety (**7**–**12**), which means that the position of the nitrogen atom influences their solubility in water. The 1,4-naphthoquinone hybrids (**19**–**24**) possess the lowest water solubility.

**Figure 3:**
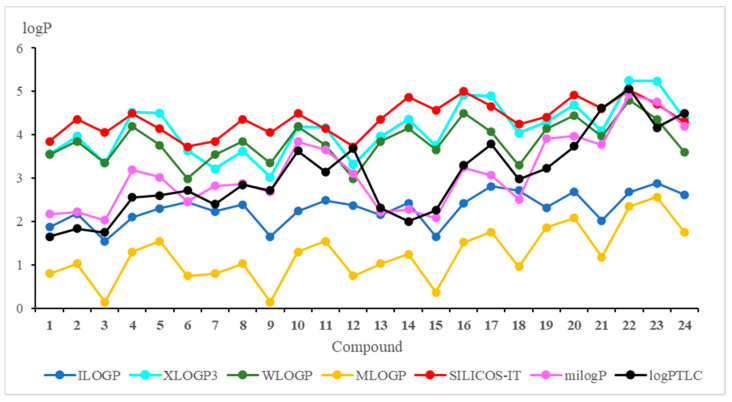
Theoretical and experimental values of the lipophilicity parameter (logP) for compounds **1**–**24**.

The theoretical lipophilicity can be evaluated by the on-line available programs [29,30,33]. The results of the theoretical approach are presented in Figure 3 and Table S1.

Figure 3 shows that the milogP program gives the logP values most similar to the experimental ones. Furthermore, it can be seen from the chemical structure of hybrids that logP depends on the substituent at the C-2 position of the quinoline moiety, and this relationship has the following order: morpholinyl ring (**6**, **12**, **18**, and **24**) > pyrrolidinyl ring (**5**, **11**, **17**, and **23**) > carbonyl group (**3**, **9**, **15**, and **21**) > hydrogen atom (**1**, **7**, **13**, and **19**) > methyl group (**2**, **8**, **14**, and **20**) > chloride atom (**4**, **10**, **16**, and **22**).

Comparison of the calculated logP values for compounds with the 5,8-quinolinedione (**1**–**6**) and 5,8-isoquinolinedione (**7**–**12**) moieties shows that the lipophilicity as determined by the WLOGP, MLOGP and SILICOS-IT programs has the same value for hybrids with the same quinoline moiety (**1** and **7**; **2** and **8**; **3** and **9**; **4** and **10**; **5** and **11**; **6** and **12**) while the experimental lipophilicities (logP_TLC_) are different. It can be concluded that, for compounds containing the 5,8-quinolinedione moiety, these programs are not suitable for calculations of lipophilicity because they do not reproduce well the experimental values.

In Table 4, the correlation equations between theoretical and experimental lipophilicity are presented. The highest correlation factor (r = 0.884) is observed for milogP program while the WLOGP program gives the worst correlation with the experiment (r = 0.417).

**Figure 4:**
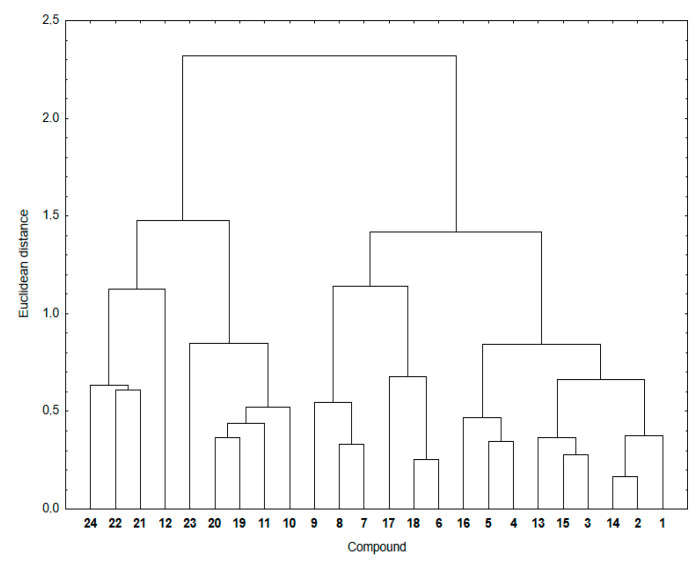
Similarity analysis for the experimental and theoretical lipophilicity for compounds **1**–**24**.

Figure 4 shows a dendrogram indicating the similarity relationship between experimental and calculated lipophilicity for compounds **1**–**24**. The theoretical lipophilicity data covers all used calculation methods.

As seen in Figure 4, the hybrids **1**–**24** are arranged in two main clusters. The first consists of the 1,4-naphthoquinone (**19**–**24**) and some 5,8-isoquinolinedione (**10**–**12**) hybrids. The second contain hybrids with the 5,8-quinolinedione (**1**–**6** and **13**–**18**) and 5,8-isoquinolinedione (**7**–**9**) moieties.

The cluster presentation is based on the Euclidean distance (ED) values [41,42,43]. The Euclidean distance is the distance in the Euclidean space of two objects whose similarity is examined by means of the similarity analysis. According to the principles of this analysis, the smaller the ED, the greater the similarity of two objects. Objects with a small ED from one another are located in the same region of the Euclidean space. To convert this distance metric to a similarity metric, we divided the object’s distance (ED) by the maximum distance in this set and then subtracted it from 1 to evaluate the similarity parameter between 0 and 1. Table 5 presents the similarity parameters for experimental and calculated lipophilicity for compounds **1**–**24**.

According to the calculation method, hybrid **12** shows a similarity parameter equal to 0, which is the smallest possible. It can be seen that, for most compounds, the similarity parameter is not very high, varying in the range 0.67–0.85. Furthermore, for highly lipophilic hybrids (**12**, **21**–**24**), the similarity parameters show the lowest values, covering the range of 0.00–0.46. Compounds with the same substituent at the C-2 position of the quinoline moiety have comparable ED distances, which means that the quinoline moiety affects the lipophilicity of hybrids.

### 3.2. ADMET Analysis

The lipophilicity is also related to other ADMET parameters such as molecular mass (MW), topological polar surface area (TPSA), number of rotatable bonds (RT), and number of acceptors (HA) and donors (HD) of the hydrogen bond. According to the rules of Lipinski and Veber, these parameters allow us to determine the bioavailability of the drug after oral administration [5,6,11,44].

As seen in Table 6, the tested hybrids meet all Lipinski rules, meaning that the molecular mass is less than 500 g/mol, and the number of donors (HD) and acceptors (HA) of hydrogen bond are less than 5 and 10, respectively. Moreover, the experimental lipophilicity is less than 5 (Table 4). The TPSA and RT of hybrids **1**–**24** are in the range 56.26–86.22 and 2–3, respectively. According to Veber’s rule, these compounds should be well absorbed orally.

**Figure 5:**
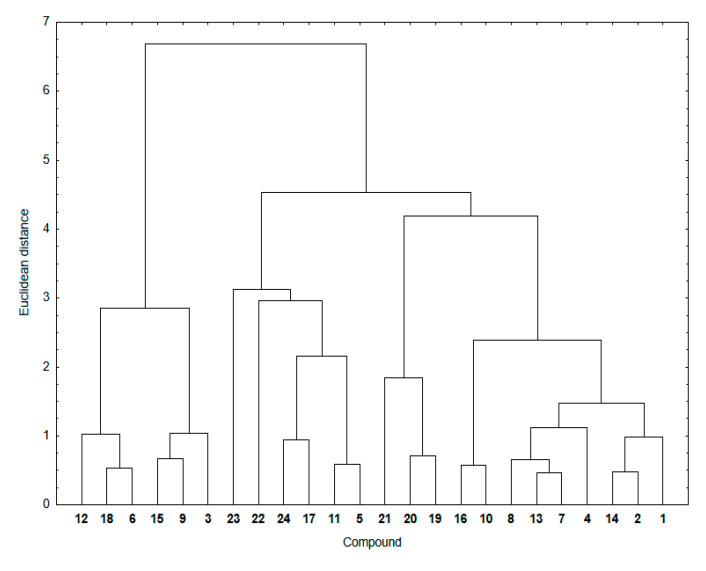
Similarity analysis for the ADMET parameters and experimental lipophilicity for compounds **1**–**24**.

Similarity analysis was used to examine a relationship between the ADMET parameters mentioned above and experimental lipophilicity for hybrids **1**–**24**. In Figure 5, the cluster analysis dendrogram showing similarities between these two sets of data is presented.

The dendrogram of the similarity analysis shows two main clusters (Figure 5). The first includes the 5,8-quinolinedione hybrids with the morpholinyl ring (**6**, **12** and **18**) and carbonyl group (**3**, **9** and **15**) at the C-2 position of the quinoline ring. The second cluster is divided into three subclusters (Figure 5). The first subcluster consists of compounds with the pyrrolidinyl ring (**5**, **11**, **17** and **23**) at the C-2 position of the quinoline ring and compounds with the 1,4-naphthoquinone moiety (**22** and **24**). The second includes the 1,4-naphthoquinone compounds (**19**–**21**). The third consists of compounds with the 5,8-quinolinedione (**1**–**2**, **4**, **13**–**14**, and **16**) and 5,8-isoquinolinedione (**7**–**8**, and **10**) moiety. As before, the similarity parameters were calculated and collated in Table 7.

It was found that, for most hybrids, the similarity parameters are high, ranging around 0.70–0.85. This means that there is a significant similarity between ADMET parameters—which can correlate with the descriptors of bioavailability—and lipophilicity of the hybrids. The exception are hybrids with high lipophilicity, for which the similarity parameters are very low, varying in the range 0.00–0.67.

In conclusion, it can be stated that, based on the similarity analysis, the relationship between ADMET parameters and experimental lipophilicity shows the lowest similarity for hybrids with the higher lipophilicity. Structural changes, such as varying the position of the nitrogen atom or substitution of the CH_3_ group, affect the lipophilicity of hybrids, and they also influence the similarity parameters in the similarity analysis. As the results so far have shown, lipophilicity can be determined experimentally or theoretically using appropriate computer programs.

The other method to determine lipophilicity (logP_calc_) is the use of ADMET parameters (Table 5). Using the Statistica program, the multilinear regression (MLR) Equation (5) has been determined, as shown below:

logP_calc_ = 0.162 TPSA − 1.200 MW + 0.674 HA + 0.310 RT + 4.431 (r = 0.721, r^2^ = 0.520, SD = 3.132, VIF = 4.19, *F* = 5.133) (5)

The lipophilicities calculated by this method for compounds **1**–**24** are summarized in Table S2. The absolute error varied in the range of 0.02–0.50. It can be noticed that there is good agreement between the lipophilicity determined in this way and the experimental one.

The bioavailability parameters influence the pharmacokinetic properties, which determine the absorption of the potential drug. Prediction of the oral and transdermal absorption was performed in silico using the Caco-2 permeability (logPapp), human intestinal absorption (HIA), and skin permeability (logKp) models. Moreover, the neurotoxicity of the compounds was designated by blood–brain barrier permeability (logBB) and central nervous system (logPS) penetration [32,44]. The pharmacokinetic parameters obtained in silico by the pkCSM software are presented in Table 8.

Lipinski and Veber descriptors are associated with pharmacokinetic parameters such as the Caco-2 permeability (logPapp) and human intestinal absorption (HIA). The compound is well absorbed and transported across the intestinal mucosa if the logPapp and HIA value are higher than 0.9 and 30%, respectively [32]. As seen in Table 6, all hybrids could be well absorbed and transmitted by the intestinal mucosa. The Caco-2 permeability (logPapp) depends on the type of the 1,4-quinone moiety and the order is as follows: 5,8-quinolinedione (**1**–**6**) > 2-methylo-5,8-quinolinedione (**13**–**18**) > 5,8-isoquinolinedione (**7**–**12**) > 1,4-naphthoquinone (**19**–**24**). The HIA index depends slightly on the type of 1,4-quinone moiety. The tested hybrids show high skin permeability because the logKp values are lower than −2.5.

One of the most important properties of a potential drug is its neurotoxicity, which is characterized by the blood–brain barrier permeability (logBB) and central nervous system penetration (logPS). The logBB values for hybrids with the 5,8-quinolinedione (**1**–**6** and **13**–**18**) and the 5,8-isoquinolinedione (**7**–**12**) moieties range from −0.671 to −1.009, which means that the compounds slowly pass through the blood–brain barrier [32]. Moreover, the logPS for compounds **1**–**18** varies from −2.045 to −2.966, which proves their poor penetration of the central nervous system [32]. Replacing the nitrogen atom with a carbon atom (**19**–**24**) causes an increase in the logBB which allows the compound to penetrate the blood–brain barrier. Similar results were obtained for logPS. For these reasons, hybrids with the 1,4-naphtoquinone moiety can be neurotoxic.

### 3.3. Quantum Chemical Descriptors

Molecular parameters, such as energy of HOMO (E_HOMO_) and LUMO (E_LUMO_) orbitals allow us to determine the global reactivity descriptors, including the ionization potential (I), electron affinity (A), hardness (η), chemical potential (µ), electronegativity (χ) and electrophilicity index (ω) [45,46,47]. These parameters can be useful for characterizing the ability of a tested compound to interact with the electrophilic and nucleophilic molecules. The energy of the HOMO and LUMO orbitals and the global descriptors are presented in Table 9.

**Figure 6:**
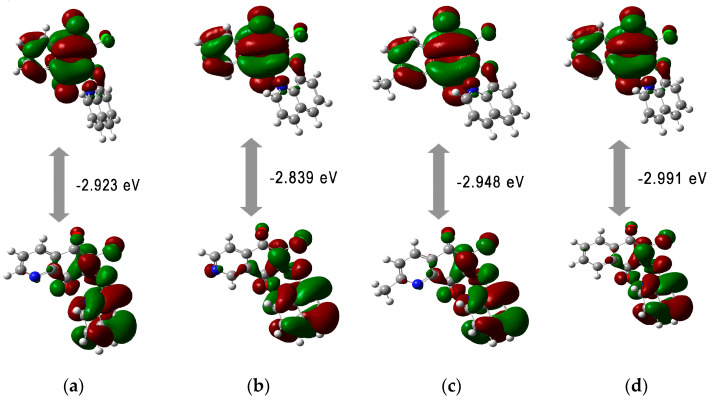
The HOMO-LUMO orbitals for compounds: (**a**) **1**; (**b**) **7**; (**c**) **13**; (**d**) **19**.

Upon analyzing the energy orbitals in relation to the molecular structure of hybrids, it can be seen that they depend on the type of the substituent at the C-2 position in the quinone moiety. Introduction of nucleophilic groups such as pyrrolidinyl (**5**, **11**, **17**, and **23**) and morpholinyl (**6**, **12**, **18**, and **24**) rings increases the energy of the HOMO orbital. However, the LUMO energy does not depend on the type of the 1,4-quinone scaffold. The HOMO orbitals are dispersed throughout the quinone scaffold and the carbonyl groups at the C-5 and C-8 positions of the 1,4-quinone moiety. The LUMO orbitals are localized at the 1,4-quinone moiety. The distribution of the HOMO and LUMO across the entire molecule indicates that the molecular system has good charge transfer capabilities (Figure 6).

All tested compounds possess comparable HOMO-LUMO energy gaps (ΔE), indicating comparable chemical reactivity. The ΔE values range from −2.045 eV to −3.240 eV showing that the hybrids **1**–**24** are characterized by high reactivity against biological targets [48]. The calculated reactivity descriptors show that hybrids have high softness and flexibility in gaining electrons. The high softness value is useful because soft drugs interact easily with an enzyme target. Moreover, the soft drug can be better metabolized into non-toxic compounds [49]. High value of electrophilicity index (ω) (7.733–11.502 eV) characterizes the tested molecules as strong electrophiles, according to the electrophilicity ranking of organic molecules [47].

The multilinear regression (MLR) Equation (6) was used to determine the enzymatic conversion rate of NQO1 (logNQO1_calc_) based on the quantum chemical properties, such as energy of LUMO (E_LUMO_) orbital and electrophilicity index (ω).

logNQO1_calc_ = 0.0500 E_LUMO_ – 0.920 ω + 0.739 (r = 0.673, r^2^ = 0.453, SD = 1.945, VIF = 2.21, *F* = 8.685) (6)

In Table S3, the enzymatic conversion rates of NQO1 (logNQO1_calc_) for hybrids **1**–**24** are collated. The absolute error varieds in the range of 0.01–0.30. The obtained results indicated that the quantum chemical descriptor could be used to determine the enzymatic conversion rate of NQO1.

### 3.4. Docking Study

According to our previous research, the tested hybrids **1**–**24** induced the mitochondrial apoptotic pathway. Molecular mechanics studies showed that hybrids reduced the number of mRNA copies of gene encoding the BCL-2 protein [22]. These results inspired the study of interactions between the ligand and the BCL-2 protein using the AutoDock Vina program [35]. Venetoclax, an inhibitor of this protein, was used as the reference substance [50].

As can be seen in Table 10, the scoring values (ΔG) obtained for hybrids **1**–**24** are lower than for venetoclax. It means that these compounds show a higher affinity for the BCL-2 protein than the reference substance. Comparing the scoring values across all compounds **1**–**24** shows that the type of 1,4-quinone affects the affinity of the ligand for the active center of the protein, and the order is as follows: 2-methyl-5,8-quinolinedione (**13**–**18**) > 1,4-naphthoquinone (**19**–**24**) > 5,8-isoquinolinedione (**7**–**12**) > 5,8-quinolinedione (**1**–**6**). It wasw found that the type of substituent at the C-2 position of the quinoline moiety affects the score value. The lowest value of ΔG was obtained for compounds with the amine substituent (**5**–**6**, **11**–**12**, **17**–**18**, and **23**–**24**), while the highest was for compounds with the hydrogen atom (**1**, **7**, **13**, **19**) at the C-2 position of the quinone moiety (Table 10).

**Figure 7:**
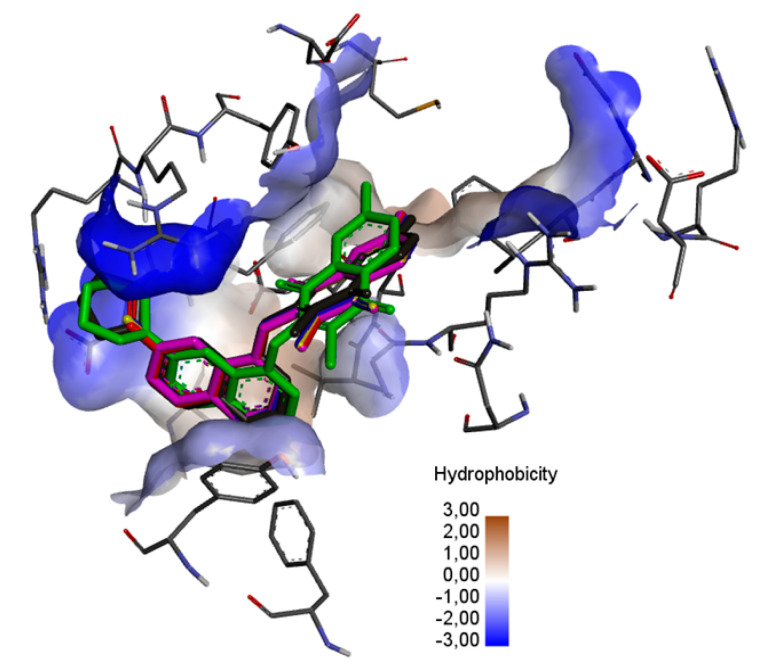
The superposition of the docked ligand: **13** (purple), **14** (blue), **15** (red), **16** (yellow), **17** (green), and **18** (grey) in the binding site of the BCL-2 protein.

Detailed analysis was performed for compounds with the 2-methyl-5,8-quinolinedione moiety (**13**–**18**). Its aim was to determine the influence of the type of quinoline substituent on the interaction with the BCL-2 protein. As can be seen in Figure 7, the ligands are localized deep within the hydrophobic matrix of the protein active center.

**Figure 8:**
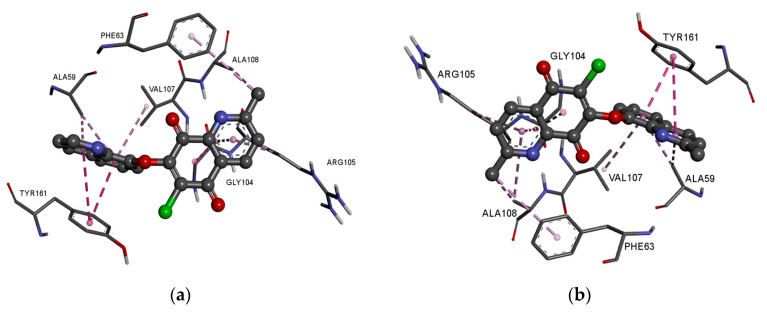

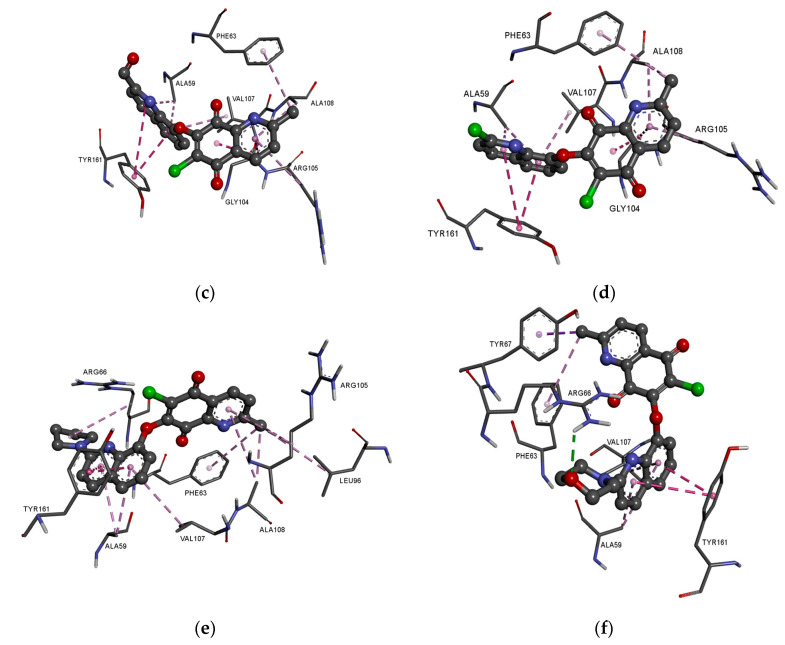
Docking pose of the BCL-2 protein complex with hybrids: (**a**) **13**; (**b**) **14**; (**c**) **15**; (**d**) **16**; (**e**) **17**; (**f**) **18**.

In the complex of ligands **13**–**16** with the BCL2 protein, the 2-methyl-5,8-quinolinedione moiety creates the hydrophobic interaction with the glycine (GLY104), arginine (ARG105), alanine (ALA105) and phenylalanine (PHE63), while the quinoline substituent interacts with the alanine (ALA59), tyrosine (THY161) and valine (VAL107) (Figure 8a–f, Table S4).

The presence of an additional amine ring (**17**–**18**) leads to a change in the arrangement of ligand in the active site of the protein (Figure 8e,f). Comparing the arrangement of **13**–**16** and **17** shows that the 2-methyl-5,8-quinolinedione and quinoline moiety create an additional hydrophobic interaction with leucine (LUE96) and arginine (ARG66), respectively (Figure 8e, Table S4). The arrangement of **18** in the active site of the protein is completely different from the others. In this case, the 2-methyl-5,8-quinolinedione interacts with phenylalanine (PHE63) and tyrosine (TYR67) via a hydrophobic interaction. The quinoline moiety interacts with alanine (ALA59), tyrosine (TYR161), and valine (VAL107). In contrast, the oxygen atom at the morpholine ring creates a hydrogen bond with arginine (ARG66) (Figure 8f, Table S4).

Comparing the arrangement of 2-methylo-5,8-quinolinedione (**13**–**18**) (Figure 8) with Venetoclax (Figure S2) shows that tested ligand interacts with similar amino acid residues in the active center of the BCL-2 protein. The hybrids **13**–**18** and reference substance interact with phenylalanine (PHE63), tyrosine (TYR67) and glycine (GLY104).

## 4. Conclusions

This research showed that the quinoline-1,4-quinone hybrids are characterized by rather low values of lipophilicity, ranging from 1.65 to 5.06. The highest values in this range were observed for hybrids containing the 1,4-naphthoquinone moiety. Introduction of the nitrogen atom reduced the lipophilicity depending on the position at the 5,8-quinolinedione moiety and this is the most important change in the structure of hybrids affecting their lipophilicity. Introduction of the nitrogen atom lowered also the hydrophobicity index describing their solubility in water. Experimental lipophilicity was compared with the theoretical values calculated by various computer programs. The milogP program reproduced the experimental lipophilicity best.

The bioavailability of the tested compounds was determined using the ADMET parameters described by the Lipinski and Veber rules. The obtained in silico parameters showed that most of the hybrids can be applied orally and that they do not exhibit neurotoxic activity. Similarity analysis was used to examine the relationship between the ADMET parameters and experimental lipophilicity. It was observed that the introduction of a nitrogen atom at the N-1 or N-2 position of the 5,8-quinolinedione moiety affects the similarity parameters, which was associated with the changes in lipophilicity of the tested hybrids.

The ability of hybrids to interact with biological targets was characterized by the global reactivity descriptors. Analysis of the descriptors showed that the compounds have high softness and can interact with nucleophilic target. Moreover, these parameters were used to determine the enzymatic conversion rate of NQO1.

The molecular docking study showed that the hybrids can inhibit the BCL-2 protein. It was also found that the type of substituent at the C-2 position of the quinoline moiety affects the scoring values.

## Figures and Tables

**Table 1:** The experimental values of R_M0_, *b*, φ_0_ and r for compounds **1**–**24**.

Compound	R_M0_	*b*	φ_0_	r	Compound	R_M0_	*b*	φ_0_	r
**1**	1.51 ± 0.02	−0.02 ± 0.01	72.87	0.974	**13**	2.09 ± 0.02	−0.03 ± 0.01	66.07	0.988
**2**	1.68 ± 0.03	−0.02 ± 0.01	68.99	0.988	**14**	1.82 ± 0.02	−0.03 ± 0.01	69.97	0.990
**3**	1.60 ± 0.03	−0.02 ± 0.01	68.09	0.978	**15**	2.05 ± 0.01	−0.03 ± 0.01	71.78	0.979
**4**	2.55 ± 0.02	−0.04 ± 0.01	65.88	0.982	**16**	2.96 ± 0.01	−0.04 ± 0.01	69.66	0.999
**5**	2.35 ± 0.02	−0.03 ± 0.01	74.53	0.990	**17**	3.39 ± 0.03	−0.05 ± 0.01	75.30	0.968
**6**	2.45 ± 0.04	−0.04 ± 0.01	66.81	0.995	**18**	2.69 ± 0.03	−0.04 ± 0.01	68.95	0.991
**7**	2.17 ± 0.01	−0.03 ± 0.01	72.57	0.996	**19**	2.90 ± 0.02	−0.04 ± 0.01	78.79	0.998
**8**	2.56 ± 0.02	−0.03 ± 0.01	74.47	0.997	**20**	3.34 ± 0.01	−0.04 ± 0.01	79.55	0.996
**9**	2.45 ± 0.02	−0.03 ± 0.01	73.71	0.998	**21**	4.11 ± 0.01	−0.05 ± 0.01	80.01	0.986
**10**	3.25 ± 0.01	−0.04 ± 0.01	78.21	0.999	**22**	4.51 ± 0.02	−0.06 ± 0.01	76.85	0.992
**11**	2.83 ± 0.03	−0.04 ± 0.01	74.76	0.999	**23**	3.72 ± 0.02	−0.05 ± 0.01	81.13	0.998
**12**	3.30 ± 0.02	−0.05 ± 0.01	72.22	0.996	**24**	4.01 ± 0.01	−0.05 ± 0.01	75.95	0.995

*b* is the slope, r is the correlation coefficient for the linear relationship R_M_ = R_M0_ + *bC*.

**Table 2:** The literature (logP_lit_) and experimental (R_M0_, *b*, and logP_TLC_, r and SD) values for the reference compounds **A**–**F**.

Substance	logP_lit_	R_M0_	*b*	r	logP_TLC_	SD
**A**	0.64	0.54	−0.02	0.991	0.54	0.052
**B**	1.21	1.11	−0.02	0.994	1.19	0.010
**C**	2.43	2.33	−0.03	0.997	2.58	0.077
**D**	3.18	2.90	−0.04	0.992	3.23	0.023
**E**	4.45	3.97	−0.05	0.993	4.45	0.002
**F**	6.38	5.60	−0.06	0.999	6.31	0.035

*b* is the slope; r is the correlation coefficient for the linear relationship R_M_ = R_M0_ + *bC*.

**Table 3:** The experimental lipophilicity (logP_TLC_) for compounds **1–24**.

Compound	logP_TLC_	Compound	logP_TLC_	Compound	logP_TLC_	Compound	logP_TLC_
**1**	1.65	**7**	2.40	**13**	2.31	**19**	3.23
**2**	1.84	**8**	2.85	**14**	2.00	**20**	3.73
**3**	1.75	**9**	2.72	**15**	2.26	**21**	4.61
**4**	2.55	**10**	3.63	**16**	3.30	**22**	5.06
**5**	2.60	**11**	3.15	**17**	3.79	**23**	4.16
**6**	2.72	**12**	3.68	**18**	2.98	**24**	4.50

**Table 4:** Correlation equations for experimental (logP_TLC_) and theoretical (logP_calc_) lipophilicity for compounds **1**–**24**.

Program	Correlation equation	r
ILOGP	logP_TLC_ = 1.524 logP_calc_ – 0.445	0.590
XLOGP3	logP_TLC_ = 0.857 logP_calc_ – 0.470	0.566
WLOGP	logP_TLC_ = 0.839 logP_calc_ – 0.162	0.417
MLOGP	logP_TLC_ = 0.994 logP_calc_ + 1.826	0.670
SILIOS-IT	logP_TLC_ = 1.051 logP_calc_ – 1.535	0.432
milogP	logP_TLC_ = 0.982 logP_calc_ – 0.007	0.884

**Table 5:** The similarity parameter (ED) for experimental and calculated lipophilicity for hybrids **1**–**24**.

Compound	ED	Compound	ED	Compound	ED	Compound	ED
**1**	0.67	**7**	0.70	**13**	0.68	**19**	0.67
**2**	0.85	**8**	0.70	**14**	0.85	**20**	0.67
**3**	0.76	**9**	0.51	**15**	0.76	**21**	0.46
**4**	0.69	**10**	0.54	**16**	0.58	**22**	0.46
**5**	0.69	**11**	0.61	**17**	0.40	**23**	0.25
**6**	0.78	**12**	0.00	**18**	0.78	**24**	0.44

**Table 6:** The Lipinski and Veber descriptors of bioavailability.

Hybrid	MW	TPSA	HA	HD	RT	Hybrid	MW	TPSA	HA	HD	RT
**1**	336.73	69.15	5	0	2	**13**	350.76	69.15	5	0	2
**2**	350.76	69.15	5	0	2	**14**	364.78	69.15	5	0	2
**3**	364.74	86.22	6	0	3	**15**	378.77	86.22	6	0	3
**4**	371.17	69.15	5	0	2	**16**	385.20	69.15	5	0	2
**5**	405.83	72.39	5	0	3	**17**	419.86	72.39	5	0	3
**6**	421.83	81.62	6	0	3	**18**	435.86	81.62	6	0	3
**7**	336.73	69.15	5	0	2	**19**	335.74	56.26	4	0	2
**8**	350.76	69.15	5	0	2	**20**	349.77	56.26	4	0	2
**9**	364.74	86.22	6	0	3	**21**	363.75	73.33	5	0	3
**10**	371.17	69.15	5	0	2	**22**	370.19	56.26	4	0	2
**11**	405.83	72.39	5	0	3	**23**	404.85	59.50	4	0	3
**12**	421.83	81.62	6	0	3	**24**	420.85	68.73	5	0	3

**Table 7:** The similarity parameter (ED) for the ADMET parameters and experimental lipophilicity for compounds **1**–**24**.

Compound	ED	Compound	ED	Compound	ED	Compound	ED
**1**	0.69	**7**	0.85	**13**	0.85	**19**	0.77
**2**	0.85	**8**	0.79	**14**	0.85	**20**	0.77
**3**	0.67	**9**	0.79	**15**	0.79	**21**	0.41
**4**	0.64	**10**	0.82	**16**	0.82	**22**	0.05
**5**	0.81	**11**	0.81	**17**	0.70	**23**	0.00
**6**	0.83	**12**	0.67	**18**	0.83	**24**	0.70

**Table 8:** Pharmacokinetic parameters of compounds **1**–**24**.

Compound	LogPapp	HIA	logKp	logBB	logPS
**1**	1.059	97.693	−2.733	−0.676	−2.047
**2**	1.234	98.041	−2.726	−0.673	−2.803
**3**	1.227	98.586	−2.740	−0.915	−2.889
**4**	1.224	96.582	−2.738	−0.849	−2.825
**5**	1.240	99.593	−2.747	−0.805	−2.911
**6**	1.291	100.000	−2.747	−0.999	−2.966
**7**	1.213	98.048	−2.728	−0.676	−2.045
**8**	1.301	98.135	−2.728	−0.671	−2.785
**9**	1.294	95.680	−2.736	−0.913	−2.871
**10**	1.291	96.677	−2.734	−0.846	−2.807
**11**	1.269	99.974	−2.743	−0.811	−2.911
**12**	1.320	100.000	−2.746	−1.006	−2.966
**13**	1.144	98.589	−2.733	−0.686	−2.803
**14**	1.243	97.942	−2.728	−0.682	−2.797
**15**	1.236	98.487	−2.742	−0.924	−2.883
**16**	1.233	96.483	−2.739	−0.858	−2.819
**17**	1.253	99.752	−2.749	−0.815	−2.902
**18**	1.304	100.000	−2.749	−1.009	−2.957
**19**	1.357	98.755	−2.735	0.187	−1.927
**20**	1.104	98.409	−2.735	0.291	−1.853
**21**	1.102	96.951	−2.737	0.278	−1.812
**22**	1.223	98.813	−2.738	−0.681	−2.133
**23**	1.122	98.886	−2.740	0.151	−1.923
**24**	1.177	100.000	−2.741	−0.772	−2.147

**Table 9:** Energy of the HOMO and LUMO orbitals, the global descriptor and enzymatic conversion rate of NQO1 for compounds **1**–**24**.

Compound	E_HOMO_ [eV]	E_LUMO_ [eV]	ΔE [eV]	I [eV]	A [eV]	η [eV]	µ [eV]	χ [eV]	ω [eV]	logNQO1 [22]
**1**	−6.522	−3.599	−2.923	6.522	3.599	1.462	−5.061	5.061	8.762	3.146
**2**	−6.448	−3.594	−2.854	6.448	3.594	1.427	−5.021	5.021	8.835	3.112
**3**	−6.689	−3.480	−3.209	6.689	3.480	1.605	−5.085	5.085	8.057	3.041
**4**	−6.750	−3.705	−3.044	6.750	3.705	1.522	−5.228	5.228	8.976	2.903
**5**	−5.684	−3.548	−2.137	5.684	3.548	1.068	−4.616	4.616	9.972	3.034
**6**	−5.855	−3.663	−2.192	5.855	3.663	1.096	−4.759	4.759	10.335	2.053
**7**	−6.629	−3.790	−2.839	6.629	3.790	1.419	−5.210	5.210	9.561	3.083
**8**	−6.553	−3.788	−2.766	6.553	3.788	1.383	−5.170	5.170	9.666	3.058
**9**	−6.568	−3.480	−3.088	6.568	3.480	1.544	−5.024	5.024	8.174	2.502
**10**	−6.614	−3.685	−2.929	6.614	3.685	1.464	−5.149	5.149	9.054	2.801
**11**	−5.775	−3.730	−2.045	5.775	3.730	1.022	−4.753	4.753	11.048	2.330
**12**	−5.932	−3.852	−2.080	5.932	3.852	1.040	−4.892	4.892	11.502	2.155
**13**	−6.474	−3.526	−2.948	6.474	3.526	1.474	−5.000	5.000	8.481	3.178
**14**	−6.404	−3.518	−2.886	6.404	3.518	1.443	−4.961	4.961	8.528	3.157
**15**	−6.643	−3.412	−3.231	6.643	3.412	1.615	−5.027	5.027	7.823	2.672
**16**	−6.702	−3.623	−3.080	6.702	3.623	1.540	−5.163	5.163	8.655	2.949
**17**	−5.640	−3.480	−2.160	5.640	3.480	1.080	−4.560	4.560	9.624	2.778
**18**	−5.810	−3.582	−2.228	5.810	3.582	1.114	−4.696	4.696	9.896	2.322
**19**	−6.465	−3.474	−2.991	6.465	3.474	1.495	−4.970	4.970	8.258	3.108
**20**	−6.392	−3.474	−2.918	6.392	3.474	1.459	−4.933	4.933	8.339	3.100
**21**	−6.626	−3.386	−3.240	6.626	3.386	1.620	−5.006	5.006	7.733	2.740
**22**	−6.693	−3.571	−3.122	6.693	3.571	1.561	−5.132	5.132	8.436	3.000
**23**	−5.636	−3.419	−2.217	5.636	3.419	1.109	−4.527	4.527	9.244	2.549
**24**	−5.796	−3.542	−2.254	5.796	3.542	1.127	−4.669	4.669	9.671	1.940

**Table 10:** Vina affinity scoring values (ΔG) [kcal/mol] for compounds **1**–**24** and venetoclax.

Compound	ΔG (kcal/mol)	Compound	ΔG (kcal/mol)
**1**	−7.50	**13**	−7.90
**2**	−7.80	**14**	−8.20
**3**	−7.70	**15**	−8.10
**4**	−7.80	**16**	−8.20
**5**	−8.20	**17**	−8.70
**6**	−8.20	**18**	−8.50
**7**	−7.70	**19**	−7.80
**8**	−7.90	**20**	−8.10
**9**	−7.90	**21**	−8.10
**10**	−7.90	**22**	−8.00
**11**	−8.10	**23**	−8.40
**12**	−8.40	**24**	−8.50
**Venetoclax**	−7.10		

## Data Availability

Not applicable.

## Supplementary Materials

The following supporting information can be downloaded at: https://www.mdpi.com/article/10.3390/pharmaceutics15010034/s1, Figure S1: The optimization structure of compounds **1**–**24**.; Figure S2. Docking pose of the BCL-2 protein complex with Venetoclax.; Table S1: Theoretical value of lipophilicity of compounds **1**–**24**.; Table S2: Experimental (logP_TLC_) and calculated (logP_calc_) value of lipophilicity and absolute error calculated by the multilinear regression (MLR) Equation (5); Table S3: Experimental (logNQO1) and calculated (logNQO1_calc_) value of lipophilicity and absolute error calculated by the multilinear regression (MLR) Equation (5) Table S4: Interaction of 2-methyl-5,8-quinolinedione hybrids **7**–**12** with BCL-2 protein.
