# Supplementary material for: Lipophilicity and ADMET Analysis of Quinoline-1,4-quinone Hybrids
Source: Pharmaceutics. 2022 Dec 22;15(1):34. doi: 10.3390/pharmaceutics15010034 (PMC9867208; doi:10.3390/pharmaceutics15010034)
Supplement: Supplementary file 1 [file pharmaceutics-15-00034-s001.zip › pharmaceutics-2068265-supplementary.pdf]

Supplementary material

Lipophilicity and ADMET analysis of quinoline-1,4-quinone hybrids

Monika Kadela-Tomanek, Maria Jastrzębska, Elwira Chrobak, Ewa Bębenek

Table of contents

Figure S1. The optimalization structure of compounds **1-24**. 2

Figure S2. Docking pose of the BCL-2 protein complex with Venetoclax. 3

Table S1. Theoretical value of lipophilicity of compounds **1-24**. 4

Table S2. Experimental ($logP_{TLC}$) and calculated by the multilinear regression (MLR) equation (5) ($logP_{calc}$) value of lipophilicity and absolute error. 4

Table S3. Experimental (logNQO1) and calculated by the multilinear regression (MLR) equation (5) ($logNQO1_{calc}$) value of lipophilicity and absolute error. 5

Table S4. Interaction of 2-methyl-5,8-quinolinedione hybrids **7-12** with BCL-2 protein. 6

Figure S1. The optimalization structure of compounds **1-24**.

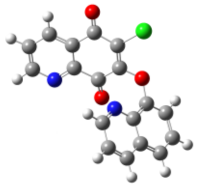

1

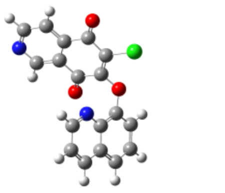

7

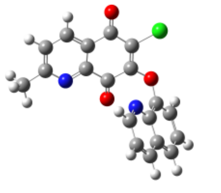

13

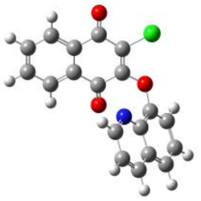

19

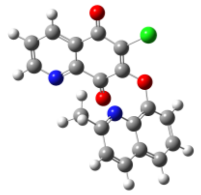

2

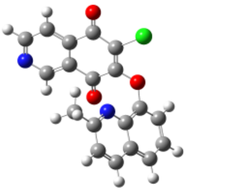

8

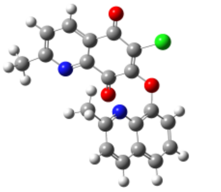

14

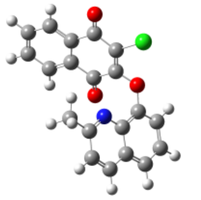

20

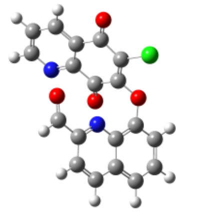

3

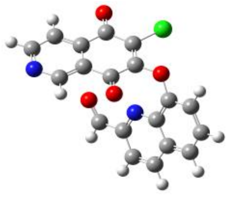

9

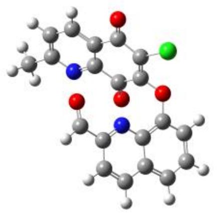

15

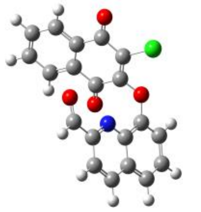

21

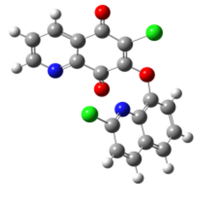

4

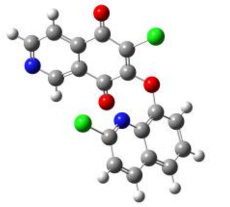

10

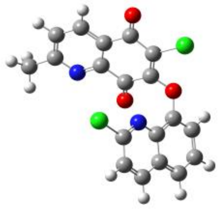

16

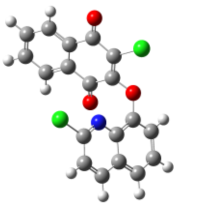

22

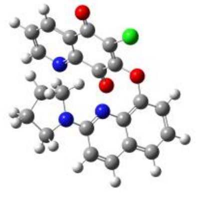

5

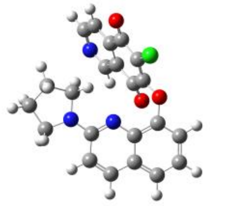

11

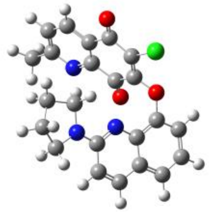

17

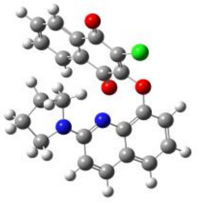

23

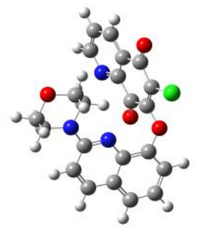

6

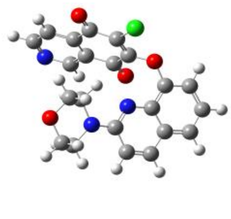

12

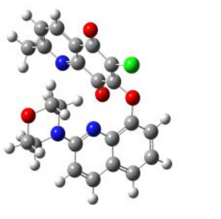

18

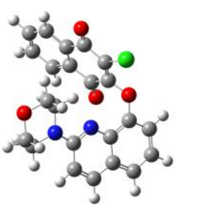

24

Figure S2. Docking pose of the BCL-2 protein complex with Venetoclax.

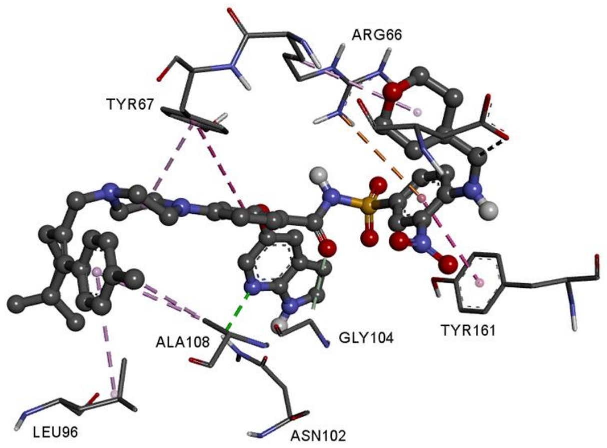

Table S1. Theoretical value of lipophilicity of compounds **1-24**.

Compound	ILOGP	XLOGP3	WLOGP	MLOGP	SILICOS-IT	milogP
**1**	1.87	3.56	3.54	0.80	3.85	2.17
**2**	2.18	3.96	3.85	1.03	4.36	2.22
**3**	1.55	3.35	3.35	0.14	4.05	2.03
**4**	2.10	4.52	4.19	1.30	4.49	3.19
**5**	2.30	4.50	3.76	1.55	4.14	3.02
**6**	2.45	3.64	2.99	0.75	3.73	2.46
**7**	2.23	3.22	3.54	0.80	3.85	2.82
**8**	2.39	3.62	3.85	1.03	4.36	2.87
**9**	1.65	3.02	3.35	0.14	4.05	2.68
**10**	2.24	4.18	4.19	1.30	4.49	3.84
**11**	2.49	4.17	3.76	1.55	4.14	3.66
**12**	2.37	3.31	2.99	0.75	3.73	3.11
**13**	2.15	3.96	3.85	1.03	4.36	2.22
**14**	2.43	4.36	4.16	1.25	4.87	2.28
**15**	1.65	3.75	3.66	0.37	4.57	2.08
**16**	2.43	4.92	4.5	1.52	5.00	3.24
**17**	2.81	4.90	4.07	1.76	4.66	3.07
**18**	2.72	4.04	3.30	0.96	4.25	2.51
**19**	2.32	4.29	4.14	1.86	4.41	3.91
**20**	2.69	4.69	4.45	2.08	4.92	3.97
**21**	2.02	4.09	3.96	1.18	4.61	3.77
**22**	2.68	5.25	4.8	2.35	5.04	4.93
**23**	2.88	5.24	4.36	2.57	4.71	4.76
**24**	2.62	4.38	3.60	1.76	4.30	4.20

Table S2. Experimental ($logP_{TLC}$) and calculated by the multilinear regression (MLR) equation (5) ($logP_{calc}$) value of lipophilicity and absolute error.

Compound	$logP_{TLC}$	$logP_{calc}$	Error	Compound	$logP_{TLC}$	$logP_{calc}$	Error
**1**	1.65	2.47	0.50	**13**	2.31	2.54	0.10
**2**	1.84	2.54	0.38	**14**	2.00	2.61	0.30
**3**	1.75	2.24	0.28	**15**	2.26	2.31	0.02
**4**	2.55	2.64	0.03	**16**	3.30	2.71	0.18
**5**	2.60	3.66	0.41	**17**	3.79	3.72	0.02
**6**	2.72	3.07	0.13	**18**	2.98	3.14	0.05
**7**	2.40	2.47	0.03	**19**	3.23	3.57	0.11
**8**	2.85	2.54	0.11	**20**	3.73	3.64	0.03
**9**	2.72	2.24	0.18	**21**	4.61	3.74	0.19
**10**	3.63	2.64	0.27	**22**	5.06	3.34	0.34
**11**	3.15	3.66	0.16	**23**	4.16	4.75	0.14
**12**	3.68	3.07	0.17	**24**	4.50	4.17	0.07

Table S3. Experimental (logNQO1) and calculated by the multilinear regression (MLR) equation (5) ($logNQO1_{calc}$) value of lipophilicity and absolute error.

Compound	logNQO1	$logNQO1_{cal}$	Error	Compound	logNQO1	$logNQO1_{cal}$	Error
**1**	3.146	2.933	0.07	**13**	3.178	2.926	0.08
**2**	3.112	2.900	0.07	**14**	3.157	2.897	0.08
**3**	3.041	3.010	0.01	**15**	2.672	2.993	0.12
**4**	2.903	3.014	0.04	**16**	2.949	3.007	0.02
**5**	3.034	2.422	0.20	**17**	2.778	2.446	0.12
**6**	2.053	2.464	0.20	**18**	2.322	2.500	0.08
**7**	3.083	2.930	0.05	**19**	3.108	2.929	0.06
**8**	3.058	2.888	0.06	**20**	3.100	2.899	0.06
**9**	2.502	2.967	0.19	**21**	2.740	2.986	0.09
**10**	2.801	2.956	0.06	**22**	3.000	3.008	0.01
**11**	2.330	2.307	0.01	**23**	2.549	2.492	0.02
**12**	2.155	2.323	0.08	**24**	1.940	2.521	0.30

Table S4. Interaction of 2-methyl-5,8-quinolinedione hybrids **7-12** with BCL-2 protein.

Ligand	2-methylo-5,8-quinolinedione moiety			Quinoline scaffold		
	Residue	Type	Distance	Residue	Type	Distance
**7**	Phe63	Pi-alkyl	4.972	Ala59	Pi-alkyl	4.476<br>4.788
	Gly104	Amide-Pi<br>Stacked	4.406<br>3.868	Val107	Pi-alkyl	5.136
	Arg105	Pi-Alkyl	4.896	Tyr161	Pi-Pi<br>Stacked	4.081<br>5.058
	Ala108	Alkyl	3.787			
**8**	Phe63	Pi-alkyl	5.015	Ala59	Pi-alkyl	4.468<br>4.651
	Gly104	Amide-Pi<br>Stacked	4.393<br>3.394	Val107	Pi-alkyl	5.091
	Arg105	Pi-Alkyl	4.852	Tyr161	Pi-Pi<br>Stacked	4.099<br>5.088
	Ala108	Pi-Alkyl	3.728<br>5.418			
**9**	Phe63	Pi-alkyl	4.986	Ala59	Pi-alkyl	4.459<br>4.668
	Gly104	Amide-Pi<br>Stacked	4.361<br>3.850	Val107	Pi-alkyl	5.074
	Arg105	Pi-Alkyl	4.865	Tyr161	Pi-Pi<br>Stacked	4.082<br>5.002
	Ala108	Alkyl	3.765<br>5.410			
**10**	Phe63	Pi-alkyl	5.004	Ala59	Pi-alkyl	4.460<br>4.633
	Gly104	Amide-Pi<br>Stacked	4.386<br>3.833	Val107	Pi-alkyl	5.072
	Arg105	Pi-Alkyl	4.852	Tyr161	Pi-Pi<br>Stacked	4.086<br>5.048
	Ala108	Alkyl	3.721<br>5.818			
**11**	Phe63	Pi-Alkyl	5.257	Ala59	Pi-Alkyl	4.573<br>4.387
	Leu96	Alkyl	5.322	Arg66	Alkyl	5.441
	Arg105	Alkyl<br>Pi-Alkyl	4.616<br>4.821	Val107	Pi-Alkyl	5.020
	Ala108	Alkyl<br>Pi-Alkyl	3.686<br>5.426	Tyr161	Pi-Pi<br>Stacked	4.098<br>5.034
**12**	Phe63	Pi-Alkyl	4.899	Ala59	Pi-Alkyl	4.694<br>4.381
	Tyr67	Pi-Sigma	3.820	Arg66	H-bond	2.527
				Val107	Pi-Alkyl	4.987
				Tyr161	Pi-Pi<br>Stacked	3.841<br>4.702
